# Effect of trans-sodium crocetinate on contrast-induced cytotoxicity in HEK-293 cells

**DOI:** 10.22038/IJBMS.2022.64671.14234

**Published:** 2023-02

**Authors:** Fatemeh Rajabian, Soghra Mehri, BiBi Marjan Razavi, Abolfazl Khajavi Rad, Mahboobeh Ghasemzadeh Rahbardar, Hossein Hosseinzadeh

**Affiliations:** 1 Department of Pharmacodynamics and Toxicology, School of Pharmacy, Mashhad University of Medical Sciences, Mashhad, Iran; 2 Pharmaceutical Research Center, Pharmaceutical Technology Institute, Mashhad University of Medical Sciences, Mashhad, Iran; 3 Targeted Drug Delivery Research Center, Pharmaceutical Technology Institute, Mashhad University of Medical Sciences, Mashhad, Iran; 4 Department of Physiology, Faculty of Medicine, Mashhad University of Medical Sciences, Mashhad, Iran; 5 Neurogenic Inflammation Research Center, Mashhad University of Medical Sciences, Mashhad, Iran

**Keywords:** Apoptosis, Autophagy, Contrast media, Cytotoxicity, Reactive oxygen species, Trans-sodium crocetinate

## Abstract

**Objective(s):**

Contrast media (CM) are used for diagnostic or therapeutic intervention purposes in medicine. The main adverse reaction after the administration of CM is contrast-induced nephropathy (CIN). This complication is the third cause of renal failure after hospital treatment. The current study is designed to investigate the possible protective effect of trans-sodium crocetinate (TSC), derived from carotenoid crocetin, against sodium amidotrizoate/meglumine amidotrizoate (SAMA) induced cytotoxicity in HEK-293 cells.

**Materials and Methods:**

HEK-293 cells were incubated with different concentrations of TSC (1, 2.5, 5, 10, 25, and 50 µM, for 48 hr) and then SAMA (7 mgI/ml, for 24 hr) was added. The cell viability, intracellular ROS, and phosphatidyl serine exposure were detected by MTT assay, DCFH-DA, and annexin V-FITC/PI method, respectively. The P-ERK/ERK ratio, apoptosis (Bax/Bcl-2 ratio and cleaved caspase-3), and autophagy (LC3 II/I ratio and beclin-1) markers in cells were evaluated by the western blot method.

**Results:**

The exposure of HEK-293 cells to SAMA reduced viability, increased apoptotic cells, enhanced ROS production, and subsequently decreased P-ERK/ERK ratio. Similarly, SAMA enhanced apoptosis (Bax/Bcl-2 ratio and cleaved caspase-3) and autophagy (LC3 II/I ratio and beclin-1) markers in HEK-293 cells. The pretreatment of cells with TSC before exposure to SAMA significantly attenuated contrast-induced cytotoxicity. TSC reduced intracellular ROS production and activated the phosphorylation of ERK. In addition, TSC decreased the levels of apoptosis and autophagy proteins.

**Conclusion:**

The pretreatment of HEK-293 cells with TSC can decrease contrast-induced cytotoxicity through antioxidant effect and modulate ERK, apoptosis, and autophagy pathways.

## Introduction

Contrast media (CM) are medical agents used to improve the visibility of internal organs and structures concerning X-ray-based imaging techniques (1). They improve the accuracy of diagnosis by enhancing the contrast between normal and pathologic areas (2). CM are used in diagnostic or interventional procedures (3, 4), especially in coronary angiography and percutaneous coronary interventions (PCI) (1). Despite the extensive use of CM, they have a significant adverse effect that leads to contrast-induced nephropathy (CIN) (2). CIN is an iatrogenic acute renal failure (3, 4) and pre-existing renal failure, diabetes mellitus, older age, and congestive heart failure are some risk factors for its incidence (5). The prevalence of CIN in the general population is 1–2% while it increases by >25% in high-risk patients (6). CIN is among the three main reasons for acute kidney disease in hospitalized patients, increased hospitalization time, chronic kidney disease, necessity for dialysis, and even mortality (6-8). The exact pathophysiological mechanisms of CIN have not been understood (9). The osmolality of CM has an effective role in the pathogenesis of CIN, so high-osmolar ionic CM are more nephrotoxic than low-osmolar nonionic CM (9). Cellular mechanisms reveal that CM enhance oxidative stress (6), inflammation (8, 10, 11), autophagy (2), and apoptosis (12). 

Oxidative stress is a direct effect of CM and it is attributed to the production of reactive oxygen species (ROS) and cytotoxicity in renal tubular epithelial and endothelial cells (6). CM exposure activates the NF-κB pathway that leads to inflammatory responses in the kidney (10), including IL-6 elevation, as the first cytokine produced after CM administration (11) and inducible nitric oxide synthase (8). CM significantly increase autophagy via the ROS-dependent pathway (2), and they enhance apoptosis via the intrinsic mitochondrial apoptotic pathway (12). 

The application of CM in medical science has been growing. Despite advances in molecular structures and the use of safer CM (low-osmolar nonionic) and preventive strategies (e.g., hydration therapy and N-acetylcysteine), CIN occurs, so a renal-protecting treatment model against CIN is needed (3, 13). 

Saffron is a traditional plant (14), the stigma of the *Crocus sativus* flower (15-17). Crocetin is one constituent of saffron (18) that has a nephroprotective effect (19). Also, different mechanisms including antioxidant (20-22), anti-inflammatory (23), anti-apoptotic (24), and regulated autophagy (15) are involved in the pharmacologic effects of crocetin.

Crocetin scavenges intracellular ROS, enhances antioxidant defense and regulates intracellular Ca^2+^ homeostasis in bovine aortic endothelial cells (20). It inhibits inflammatory cytokines, induces nitric oxide synthase (iNOS), and generates NF-κB because crocetin has a pseudo-NSAID nature (23). Crocetin diminishes all regulations that cause mitochondrial-mediated apoptosis including suppression of ERK1/2, PI3K/AKT, p38 expression, and provocation of the p53/p21 pathway in KYSE-150 cells (24). Also in the human breast cancer cell line (MCF-7 cells), it regulates autophagy via decreasing autophagic proteins (15). Crocetin in HEK-293 cells prevents ROS creation and repairs the antioxidant system. Also, it inhibits the ROS liberation from mitochondria to the cytoplasm via stabilization of the mitochondrial membrane potential (19). Therefore, crocetin can be an effective treatment for CIN inhibition.

Trans-sodium crocetinate (TSC) is derived from the carotenoid crocetin (25, 26). TSC is a bipolar trans-carotenoid salt that improves the bioavailability of crocetin and accelerates its therapeutic effects (16). Plasma is the main barrier for oxygen to adsorb and release freely from the red cells. TSC decreases the plasma resistance and increases the release of oxygen from the red cells to the tissue (26, 27), so it enhances the diffusivity of oxygen (28, 29).

Direct cytotoxic effects of CM on renal tubular cells are investigated under *in vitro* conditions because of a lack of intervening parameters such as hypoxia following intrarenal vasoconstriction and other systemic mechanisms which occur *in vivo* (9, 30). This study is designed to evaluate the protective effect of TSC on sodium amidotrizoate/meglumine amidotrizoate (SAMA) induced cytotoxicity in HEK-293 cells (Human embryonic kidney cells) with a focus on cellular and molecular mechanisms including oxidative stress, apoptosis, autophagy, and MAP kinase signaling pathways.

## Materials and Methods

***Materials***

Sodium amidotrizoate/meglumine amidotrizoate (SAMA), also known as Urografin 76% was used as a contrast medium and purchased from Bayer Schering Pharma (Germany). The concentrations of sodium amidotrizoate and meglumine amidotrizoate were 0.1 g/ml and 0.66 g/ml, respectively, so it contained 0.37 g iodine/ml. Fluorescent probe 2, 7-dichlorofluorescein diacetate (DCF-DA) and 3-(4,5-dimethylthiazol-2-yl)-2,5-diphenyl tetrazolium (MTT) were obtained from Sigma (Munich, Germany). Dulbecco’s modified Eagle’s medium (DMEM) high glucose and fetal bovine serum (FBS) were prepared from Gibco. The polyvinylidene fluoride (PVDF) membrane was acquired from Bio-Rad (CA, USA). Trans-sodium crocetinate (TSC) and N-acetylcysteine (NAC) were obtained from Tinab Shimi (Mashhad, Iran). Annexin V-FITC/PI apoptosis detection kit was purchased from Zist Pajohan Mahbob Company (Tehran, Iran).

***Cell culture***

In the current study, HEK-293 cells (human embryonic kidney cells) were provided by the Pasteur Institute (Tehran, Iran). In brief, cells were cultured in DMEM high glucose supplemented with 10 vol% of heat-inactivated FBS, 100 U/ml penicillin, and 100 µg/ml streptomycin. Cells were maintained in a humidified atmosphere (90%) at 37 ^°^C containing 5% CO_2_ in the air (31).

***Cell viability***

The cell viability was evaluated using the MTT assay (32). In brief, HEK-293 cells were cultured in 96-well microplates (2500 cells/well). Then the cells were incubated for 24 hr in a growth medium (DMEM) at 37 ^°^C. To evaluate the cytotoxic effect of SAMA on HEK-293 cells, they were treated with different concentrations of SAMA (1.5-185 mgI/ml), incubated for 24 hr, and the cell viability was determined by MTT assay. The concentration of SAMA that causes 50% of cell death (IC50) was assessed using the GraphPad Prism 8 statistical software. Furthermore, to study the cytoprotective effect of TSC, cells were pretreated with nontoxic concentrations of TSC (1, 2.5, 5, 10, 25, and 50 µM) and incubated for 48 hr. Then SAMA at a concentration of 7 mgI/ml was added and incubated for another 24 hr. NAC (50 µM) was used as a positive control (33, 34). Finally, MTT solution (concentration 0.5 mg/ml) was added to the cells and incubated at 37 ^°^C. After 3 hr the medium was eliminated and the purple formazan crystals were dissolved in 100 µl dimethylsulfoxide (DMSO). Absorbance was measured at 545 and 630 nm in an ELISA reader (Start Fax-2100, UK). 

***Measurement of intracellular ROS generation***

The formation of intracellular ROS was detected by the DCFH-DA method (35). DCFH-DA (nonfluorescent) is passed across cell membranes and is then hydrolyzed to DCFH (nonfluorescent) by intracellular esterases. Intracellular ROS converted DCFH to highly fluorescent dichlorofluorescein (DCF). Briefly, HEK-293 cells ( 2500 cells/well) were cultured in 96-well plates and incubated for 24 hr. Different concentrations of TSC (1, 2.5, 5, 10, 25, and 50 µM) were used to pre-treat the cells for 48 hr. After that, SAMA (7 mgI/ml) was added and incubated for another 24 hr. Finally, the medium was eliminated and cells were washed with PBS twice and incubated with 10 μM of DCFH-DA (30 min, at 37 ^°^C). In a microplate reader, the fluorescence intensity of DCF was measured at 485 nm (excitation wavelength) and 528 nm (emission wavelength).

***Annexin V-fluorescein isothiocyanate conjugated (FITC)/propidium iodide (PI) staining test***

This test measured apoptosis via phosphatidylserine exposure (36). The HEK-293 cells (70000 cells/well) were cultured in a 12-well plate and were incubated with TSC (1 and 5 µM) and NAC (50 µM), as the positive control, for 48 hr. Then the cells were exposed to SAMA (7 mgI/ml) and after 24 hr, the supernatant was discarded and PBS was added to wash the cells. The cells were detached with trypsin-EDTA and were centrifuged at 1500 rpm for 5 min. The supernatant was removed and the cells were washed with PBS. The microtubes were centrifuged and the supernatant was removed. The cells were resuspended in 500 µl of 1X binding buffer and 2 µl of Annexin V-FITC conjugate were added, then they were incubated for 10–15 min at room temperature in the dark. Finally, 2 µl of the PI solution was added to the cell suspension and was incubated 1–5 min at room temperature with protection from light. Finally, the fluorescein-5-isothiocyanate (FITC) and PI fluorescence of each sample was measured using the BD Accuri™ C6 flow cytometer (USA).

***Western blot analysis***

Using the western blot technique, the apoptosis pathway proteins (Bax, Bcl-2, and caspase 3), autophagy pathway proteins (beclin-1 and LC3 II/I), and the proteins of the MAP kinase signaling pathway (ERK1/2 and phospho-ERK) were determined. HEK-293 cells were cultured in T-75 flasks (106 cells/T-75). After treatment of cells with TSC (1 and 5 µM) and SAMA (7 mgI/ml) cells were harvested and washed with cold PBS and then were lysed by a lysis buffer containing the following: complete protease inhibitor cocktail, 2 mM EDTA, 50 mM Tris-HCl (pH: 7.4), 2 mM EGTA, 1 mM sodium orthovanadate (Na3VO4), 10 mM NaF, 10 mM beta-glycerophosphate, 0.2% W/V sodium deoxycholate, and 1 mM phenylmethylsulfonyl fluoride (PMSF). The cells were centrifuged at 4000 g for 10 min at 4 °C, then the supernatant was collected. The protein content of supernatants was measured by Bradford assay. Supernatants in equal amounts of total proteins were loaded and electrophoresed in 12% SDS-PAGE gels and transferred to PVDF membranes. 5% skim milk and 5% bovine serum albumin were used to block the membranes respectively for non-phosphorylated and phosphorylated proteins for 2 hr at room temperature. Then blots were incubated with primary antibodies, including rabbit polyclonal anti-serum against Bax (Cell Signaling, #2772), rabbit monoclonal anti-serum against Bcl-2 (Cell Signaling, #2870), rabbit monoclonal anti-serum against caspase-3 (Cell Signaling, #9665), rabbit monoclonal anti-serum against cleaved caspase-3 (Cell Signaling, #9664), rabbit monoclonal anti-serum against ERK1/2 (Cell Signaling, #4695), rabbit monoclonal anti-serum against phospho-ERK1/2 (Cell Signaling, #4370), rabbit monoclonal anti-serum against beclin-1 (Cell Signaling, #3495), rabbit monoclonal anti-serum against LC3 II/I (Cell Signaling, #12741), and mouse monoclonal anti-serum against beta-actin (Cell Signaling, #3700) at 1:1000 dilutions for 16-18 hr at 4 ^°^C. The membranes were washed three times with TBST. They were incubated with anti-rabbit IgG labeled with horseradish peroxidase (Cell Signaling, #7074) or anti-mouse IgG labeled with horseradish peroxidase (Cell Signaling, #7076) at 1:3000 dilutions for 1.5 hr at room temperature. Finally, the protein bands were detected by applying an enhancing chemiluminescence reagent and measuring the optical densities of the bands using Alliance 4.7 Geldoc (UK). Densitometric analysis of the protein bands was done using UVtec software (UK). The normalization of protein bands was done against beta-actin (36). 

***Statistical analysis***

All results are expressed as mean±standard deviation (SD). Statistical analyses were performed using one-way ANOVA following the Tukey-Kramer post-test for comparison of means (Prism 8.0, GraphPad Software Inc., CA, USA). Differences were considered statistically significant when *P* was less than 0.05.

## Results

***Effect of contrast medium on cell viability in HEK-293 cells***

**Figure 1:**
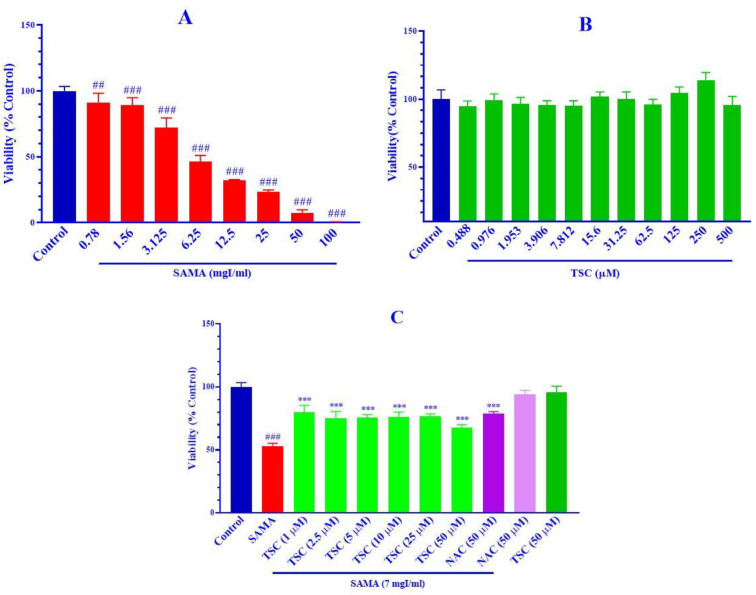
A) Effect of SAMA (0-100 mgI/ml), B) TSC (0-500 µM) on HEK-293 cells, C) Protective effect of TSC (1, 2.5, 5, 10, 25, and 50 µM) and NAC (50 µM) on contrast-induced toxicity on HEK-293 cells. Cell viability was determined by MTT assay. Data are shown as mean±SD of four separate experiments. Data were analyzed by one-way ANOVA following the Tukey-Kramer post-test. ^###^*P<*0.001 and ^##^*P<*0.01 vs control group, ***P<0.001 vs SAMA treated cells

SAMA: Sodium amidotrizoate/meglumine amidotrizoate; TSC: Trans-sodium crocetinate; NAC: N-acetyl cysteine

The cytotoxicity of SAMA in HEK-293 cells was evaluated using an MTT assay. Figure 1A shows that the exposure of HEK-293 cells to SAMA for 24 hr decreased significantly the cell viability in a concentration-dependent manner compared with the control group. The IC_50_ value (50% inhibitory concentration) of SAMA was calculated at 6.66±0.02 mgI/ml. 

***Effect of TSC on cell viability in HEK-293 cells***

TSC in the different concentrations of 0-500 µM for 48 hr, had no cytotoxic effect on HEK-293 cells in comparison with the control group, as exhibited in Figure 1B.

***Effect of TSC on contrast-induced cytotoxicity in HEK-293 Cells***

The exposure of HEK-293 cells to SAMA (7 mgI/ml) for 24 hr, significantly decreased cell viability in comparison with the control group (*P*<0.001). The pretreatment of cells with TSC (1, 2.5, 5, 10, 25, and 50 µM) and NAC (50 µM) significantly decreased cytotoxicity compared with the SAMA group (*P*<0.001). TSC (50 µM) was not cytotoxic against HEK-293 cells (Figure 1C).

***Effect of TSC on contrast-induced ROS production in HEK-293 Cells***

**Figure 2:**
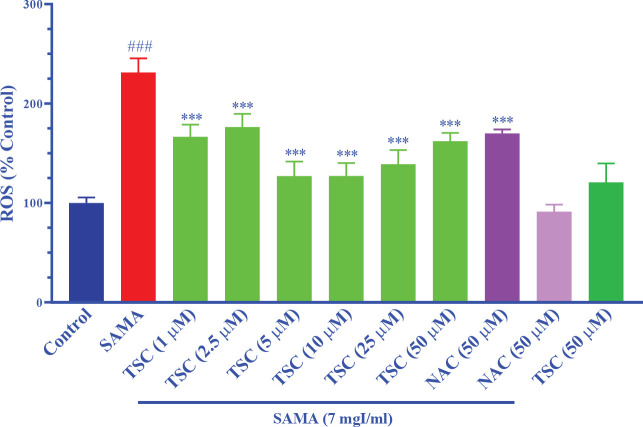
Effect of TSC on contrast-induced ROS generation in HEK-293 cells

The results in Figure 2 show that SAMA (7 mgI/ml) significantly enhanced ROS production after 24 hr exposure in comparison with the control group (*P*<0.001). Pretreatment with 1, 2.5, 5, 10, 25, and 50 µM of TSC and NAC (50 µM) significantly reduced the levels of intracellular ROS compared with the SAMA group (*P*<0.001). TSC (50 µM) and NAC (50 µM) did not increase the levels of intracellular ROS in comparison with the control group.

***Effect of TSC on SAMA-induced apoptosis in HEK-293 cells***

**Figure 3:**
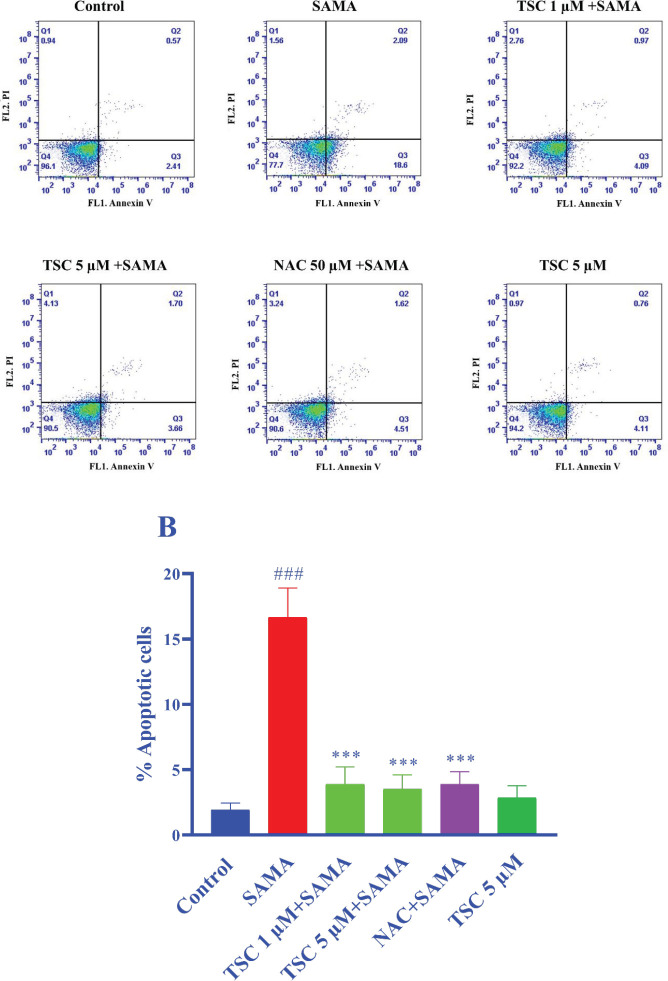
Effect of TSC and NAC on apoptosis induced by SAMA in HEK-293 cells. Flow cytometry detection of apoptosis with annexin V-FITC/PI test

The percentage of apoptotic cells was determined using Annexin V-FITC/PI staining test. Exposure to SAMA (7 mgI/ml) markedly enhanced apoptotic cells in HEK-293 cells in comparison with the control (*P*<0.001). Pre-treatment of cells with TSC (1 and 5 µM) significantly reduced the number of apoptotic cells compared with the control group (*P*<0.001). NAC (50 µM), as a positive control, also decreased the number of apoptotic cells (*P*<0.001 vs control group). Treatment with TSC (5 µM) alone did not show any apoptosis effect (Figures 3A & B).

***Effect of contrast medium and TSC on the level of apoptosis pathway proteins in HEK-293 Cells***

**Figure 4:**
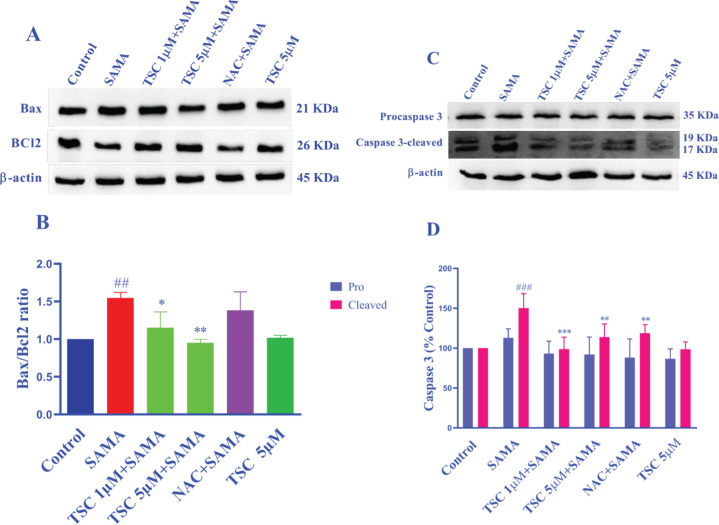
Effect of SAMA, TSC, and NAC on Bax/Bcl-2 ratio and caspase-3 (pro and cleaved) proteins in HEK-293 cells

Apoptosis was evaluated by measuring the Bax/Bcl-2 ratio and caspase-3 expression in HEK-293 cells. As shown in Figure 4, exposure of HEK-293 cells to SAMA (7 mgI/ml) for 24 hr significantly enhanced the Bax/Bcl-2 ratio (*P*<0.01 vs control) and cleaved caspase-3 (*P*<0.001 vs control). However, pretreatment with 1 and 5 µM of TSC for 48 hr, in cells exposed to SAMA, reduced the Bax/Bcl-2 ratio (*P*<0.05 and *P*<0.01, respectively) and cleaved caspase-3 (*P*<0.001 and *P*<0.01, respectively) in comparison with the SAMA group. Pretreatment of cells with NAC (50 µM) reduced the expression of cleaved caspase-3 compared with the SAMA group (*P*<0.01).

***Effect of TSC and contrast medium on the expression of ERK and P-ERK proteins in HEK-293 cells***

**Figure 5:**
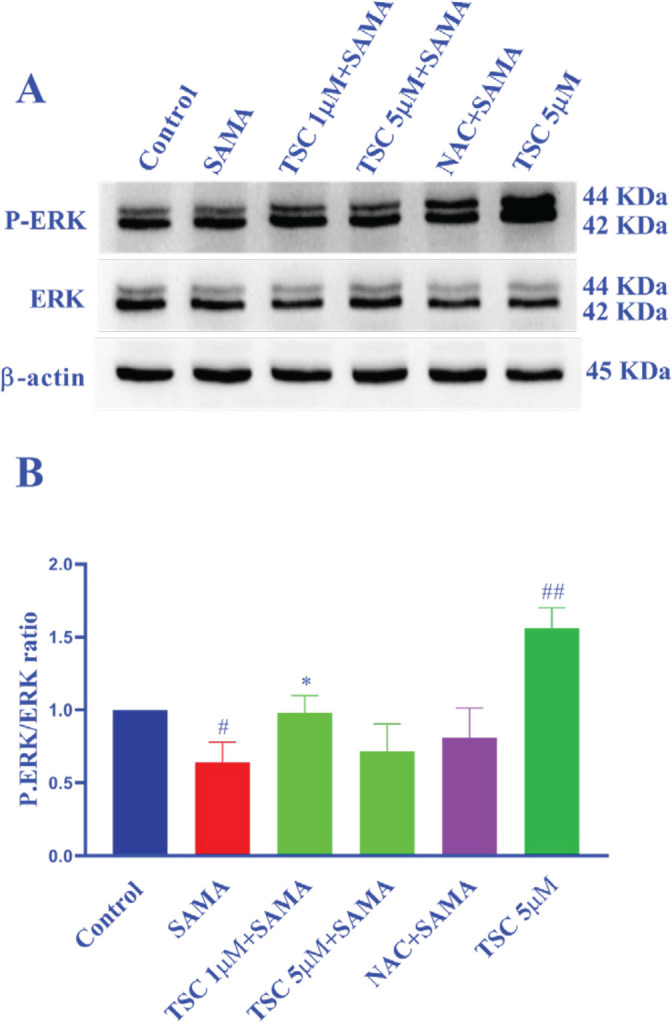
Effect of SAMA, TSC, and NAC on the ratio of P-ERK/ERK in the HEK-293 cells through western blotting analysis

Treatment of HEK-293 cells with SAMA at a concentration of 7 mgI/ml, significantly decreased the P-ERK/ERK ratio compared with the control group (*P*<0.05). On the other hand pretreatment of cells with TSC (1 µM) increased the P-ERK/ERK ratio when compared with the SAMA group (*P*<0.05). Also, TSC 5 µM significantly increased the P-ERK/ERK ratio in comparison with the control group (*P*<0.01) (Figure 5).

***Effect of contrast medium and TSC on autophagy proteins in HEK-293 cells***

**Figure 6:**
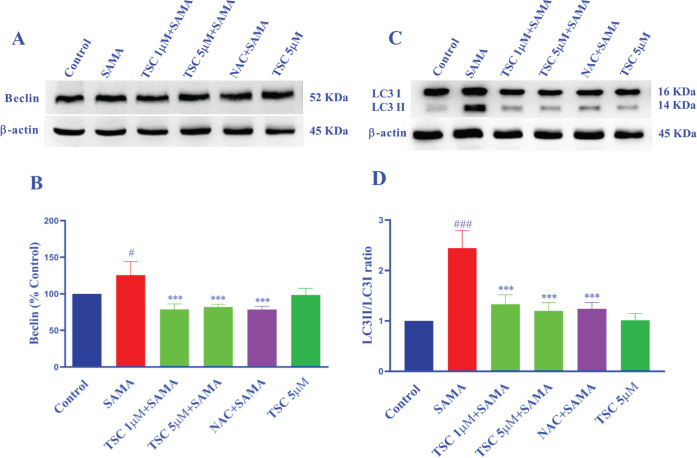
Effect of SAMA, TSC, and NAC on the protein expressions of beclin-1 and LC3 II/I ratio in HEK-293 cells

The expression levels of LC3 and beclin-1 as markers of the autophagy pathway were determined by western blot (Figure 6). Following exposure of HEK-293 cells to SAMA (7 mgI/ml) for 24 hr, beclin-1 (*P*<0.05) and LC3-II/I ratio (*P*<0.001) significantly increased compared with the control group. Pre-treatment of cells with TSC (1 and 5 µM) significantly decreased beclin-1 (*P*<0.001) and LC3-II/I ratio (*P*<0.001) in comparison with the SAMA group. Also, NAC (50 µM) significantly decreased the expression of beclin-1 (*P*<0.001 vs SAMA) and LC3-II/I ratio (*P*<0.001 vs SAMA).

## Discussion

In the present study, we evaluated the protective effect of TSC on contrast-induced cytotoxicity in HEK-293 cells via several intracellular signaling pathways. The exposure of cells to SAMA for 24 hr reduced viability, elevated ROS production, and the number of apoptotic cells and subsequently decreased the P-ERK/ERK ratio in HEK-293 cells. Also, SAMA enhanced the apoptosis (Bax/Bcl-2 ratio and cleaved caspase-3) and autophagy (beclin-1 and LC3II/I ratio) markers in cells. The pretreatment of HEK-293 cells with TSC and NAC as a positive control, for a period of 48 hr, suppressed contrast-induced cytotoxicity, ROS production, and apoptosis. Moreover, TSC pretreatment increased the P-ERK/ERK ratio and decreased apoptosis and autophagy markers against SAMA cytotoxicity.

The application of CM in clinical practice is for diagnostic and interventional procedures (37). Therefore management of CIN as the major adverse effect of CM is really important. Diatrizoate is an iodine contrast medium that has a high osmolality (37). According to *in vitro* studies, incubation of HK-2 cells (human renal proximal tubular epithelial cell line) to sodium diatrizoate (75 mg/ml, for 2 hr) decreased cell viability (38, 39). In our study, SAMA (0–100 mgI/ml, for 24 hr) concentration-dependently decreased the viability of HEK-293 cells.

Exposure to CM enhances cellular sensitivity against oxidative stress (40) because they elevate intracellular ROS (41) or declines antioxidant enzymes (40). Iodine atoms of CM can produce oxygen radicals (42) which lead to direct cellular injury (41), and finally, enhance intracellular ROS in HEK-293 cells (19). In this research, following exposure of HEK-293 cells to SAMA (7 mgI/ml, for 24 hr), ROS production was increased. 

TSC is a strong antioxidant that scavenges free radicals (29), mainly hydroxyl radicals (25), and increases the endogenous antioxidant enzymes, and finally declines intracellular ROS (19). Pre-treatment of bovine aortic endothelial cells with crocetin (1 µM, for 12 hr) showed that it decreased intracellular ROS generation (20). Crocetin and its nanoformulation (0.1, 0.5, and 1 μM, for 24 hr) increased endogenous antioxidant enzymes, so they detoxified the free radicals in cyclosporine A-mediated cytotoxic in HEK-293 cells (19). In line with previous reports, we found out that ROS production had a main role in SAMA toxicity in HEK-293 cells. We also indicated that the pre-incubation of cells with TSC (1, 2.5, 5, 10, 25, and 50 µM) for 48 hr enhanced viability and reduced the generation of ROS.

Extracellular signal-regulated kinase 1/2 (ERK) belongs to the mitogen-activated protein kinase (MAPK) family and regulates cell stress responses, differentiation, and proliferation (43). 

ROS activates the ERK pathway (44) which subsequently phosphorylates Bim and/or Bad. Consequently, the cells’ sensitivity towards apoptosis is decreased (24). Andreucci *et al*. (2011), showed that diatrizoate (75 mgI/ml, for 2 hr) lowered the phosphorylation of the ERK1/2 kinases in HK-2 cells (38). Also in another study, sodium diatrizoate (75 mgI/ml) significantly decreased the phosphorylation status of ERK and diminished the growth and proliferation of HK-2 cells (39). According to previous research, our results indicated that SAMA (7 mgI/ml, for 24 hr) significantly decreased the P-ERK/ERK ratio in HEK-293 cells. Crocetin pretreatment (5 µM, for 24 hr) activated the phosphorylation of ERK-1/2 via its anti-oxidative effect and had a neuroprotective effect against Aβ_1-42 _toxicity in Ht22 cells (45). 

The results of the current research demonstrated that pre-treatment with TSC at the concentration of 1 μM for 48 hr significantly enhanced the P-ERK/ERK ratio in HEK-293 cells.

According to reports, in contrast-induced kidney damage, the incidence of oxidative stress and subsequent apoptosis were important in cells (40). Apoptosis is an imperative mechanism in the pathogenesis of CIN in renal tubular cells (4). Several studies reported that CM cause apoptosis in cells (4, 46-48). CM (amidotrizoate, 100 mgI/ml, 30 min) enhanced the percentage of apoptotic cells which was shown by increased number of stained cells with Annexin V-FITC/PI (48). In another study, exposure to CM (Iohexol, 20-40 mgI/ml, for 72 hr) led to cell apoptosis in a concentration-dependent manner in HK-2 cells which was determined using Annexin V-FITC/PI test (49). We indicated SAMA (7 mgI/ml) after 24 hr exposure increased the number of apoptotic cells in HEK-293 cells. 

Zhang *et al*. (2020) showed that radiation on rat intestinal epithelial IEC-6 cells increased the percentage of apoptotic cells while crocetin (10 μM) decreased radiation-induced apoptosis (50). In this study, TSC and NAC significantly decreased SAMA-induced apoptosis in HEK-293 cells.

Furthermore, Jiang *et al*. revealed that meglumine diatrizoate (100 mg/ml) increased the expression of caspase-3 and Bax while it decreased the level of Bcl-2 (apoptosis inhibitor) in HK-2 cells (47). Also, iopamidol in both *in vivo* (2.9 gI/kg in rats) and *in vitro* (200 mg/ml on HK-2 cells) models elevated the levels of Bax/cleaved caspase-3 and declined the level of Bcl-2 (4). Similar to previous research, we showed that exposure of HEK-293 cells to SAMA (7 mgI/ml) for 24 hr enhanced the levels of Bax/Bcl-2 ratio and cleaved caspase-3.

The protective role of crocetin against cellular apoptosis has been reported in several studies. It increases antioxidant activity and modulates intracellular calcium homeostasis (20). Crocetin stabilizes the mitochondrial membrane potential (19) and enhances the inherent mitochondrial and cytosolic toleration against apoptotic triggers (51).

Liu *et al*. showed the protective effect of crocetin in arsenic trioxide-induced renal injury in rats by reducing apoptosis. Crocetin declined caspase-3 and Bax and also enhanced Bcl-2 (51). Myocardial ischemia/reperfusion damage in rats induced apoptosis in the heart, while administration of TSC exhibited cardioprotective properties through anti-apoptosis effects (52). In the present study, pre-treatment of HEK-293 cells with TSC (1 and 5 μM, for 48 hr) decreased the level of Bax/Bcl-2 ratio and cleaved caspase-3.

Autophagy is a cellular response to lysosome-triggered intracellular degradation of damaged proteins or organelles and reuses nutrients for cell survival (53). It may have a protective role against cell damage and reduces cell apoptosis (7). According to experiments, in acute kidney damage, the immediate function of autophagy has a protective role in cells (47, 54). Following CM exposure, organelles are damaged, and the lysosomes are enhanced, consequently, autophagy is activated and may improve renal cell survival (7). A study showed that meglumine diatrizoate (100 mg/ml) enhanced the expression of autophagy markers (beclin-1 and LC3 II/LC3 I ratio) in HK-2 cells (47). Also, an *in vitro* study revealed that after exposure of HK2 cells to iohexol (200 mgI/ml, for 6 hr), the expression of LC3II was elevated (7). We showed that SAMA (7 mgI/ml, for 24 hr) increased the LC3 II/I ratio and beclin-1 in HEK-293 cells.

Crocetin was effective in breast cancer cells (MCF-7) through regulation of the autophagy pathway (decreased the level of beclin-1) (15). In the current study, TSC pretreatment (1 and 5 μM) declined the LC3 II/I ratio and beclin-1 in HEK-293 cells.

Data are expressed as mean±SD of three separate experiments. Data were analyzed by one-way ANOVA following the Tukey-Kramer post-test. ^###^*P<*0.001 vs control group and ***P<0.001 vs SAMA group. SAMA: Sodium amidotrizoate/meglumine amidotrizoate; TSC: Trans-sodium crocetinate; NAC: N-acetyl cysteine

A) Flow cytometry plots with annexin V-FITC/PI in different groups and B) Column bar graph for the percentage of apoptotic cells in different groups. Data are shown as the mean±SD of three separate experiments. ^###^*P<*0.001 vs control group and ***P<0.001 vs SAMA group

SAMA: Sodium amidotrizoate/meglumine amidotrizoate; TSC: Trans-sodium crocetinate; NAC: N-acetyl cysteine

A) Western blot bands of Bax, Bcl-2, and beta-actin, C) Western blot bands of procaspase 3, cleaved caspase-3 and beta-actin, B and D) Densitometry analysis of western blots for the Bax/Bcl-2 ratio and caspase-3 (pro and cleaved) protein levels after normalization with beta-actin. Data are expressed as means±SD of three independent experiments. Data were analyzed by one-way ANOVA following the Tukey-Kramer post-test. ^##^*P<*0.01 and ^###^*P<*0.001 vs control group, *P<0.05, **P<0.01, and ***P<0.001 vs SAMA group

SAMA: Sodium amidotrizoate/meglumine amidotrizoate; TSC: Trans-sodium crocetinate; NAC: N-acetyl cysteine

A) Western blot bands of P-ERK, ERK, and beta-actin, B) Densitometry analysis of western blots for P-ERK and ERK proteins. Data are shown as mean±SD for three independent experiments. Data were analyzed by one-way ANOVA following the Tukey-Kramer post-test. ^#^*P<*0.05 and ^##^*P<*0.01 vs control group, *P<0.05 vs SAMA group

SAMA: Sodium amidotrizoate/meglumine amidotrizoate; TSC: Trans-sodium crocetinate; NAC: N-acetyl cysteine

A) Western blot bands of beclin-1 and beta-actin, C) Western blot bands of LC3 II/I ratio and beta-actin, B and D) The densitometry analysis of western blots for beclin-1 and LC3 II/I ratio after normalization with beta-actin. Data are expressed as mean±SD for three independent experiments. Data were analyzed by one-way ANOVA following the Tukey-Kramer post-test. ^###^*P<*0.001, #*P<*0.05 vs control group and ***P<0.001 vs SAMA group

SAMA: Sodium amidotrizoate/meglumine amidotrizoate; TSC: Trans-sodium crocetinate; NAC: N-acetyl cysteine

**Figure 7:**
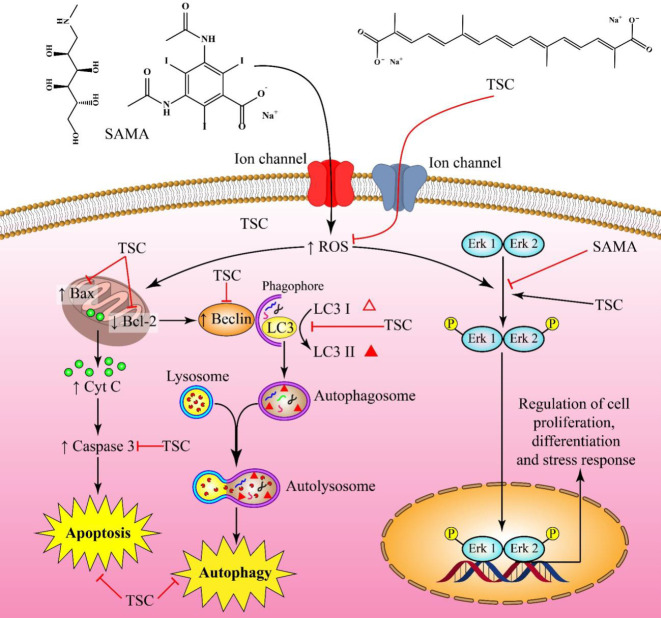
Protective effect of TSC on SAMA-induced cytotoxicity in HEK-293 cells through oxidative stress, apoptosis, autophagy, and MAP kinase signaling pathways. The black arrows indicate the stimulatory effects of SAMA and TSC. The red arrows demonstrate the inhibitory effects of TSC and SAMA

SAMA: Sodium amidotrizoate/meglumine amidotrizoate; TSC: Trans-sodium crocetinate; Cyt C: Cytochrome C; ROS: Reactive oxygen species; ERK: Extracellular signal-regulated kinase

## Conclusion

SAMA leads to cytotoxicity in HEK-293 cells by elevating oxidative stress and decreasing P-ERK/ERK ratio. Furthermore, SAMA-induced apoptosis (number of apoptotic cells, Bax/Bcl-2 ratio, and caspase-3) and autophagy (LC3 II/I ratio, beclin-1) markers. TSC shows a protective effect against contrast-induced cytotoxicity via the anti-oxidant effect, activation of the phosphorylation of ERK, anti-apoptosis properties, and modulation of the autophagy pathway (Figure 7).

## Authors’ Contributions

FR performed experiments, collected the data, and wrote the manuscript; SM, BBMR, AKR, and MGR were the co-supervisors; FR and SM analyzed the data, evaluated and interpreted the results; HH was the principal investigator for the project; SM and HH planned the main idea of the research, organized and led the project. All authors approved the current version of the manuscript.

## Conflicts of Interest

The authors declare that there are no conflicts of interest.
